# FGF-induced *LHX9* regulates the progression and metastasis of osteosarcoma via FRS2/TGF-β/β-catenin pathway

**DOI:** 10.1186/s13008-019-0056-6

**Published:** 2019-11-25

**Authors:** Shuang-Qing Li, Chao Tu, Lu Wan, Rui-Qi Chen, Zhi-Xi Duan, Xiao-Lei Ren, Zhi-Hong Li

**Affiliations:** Orthopaedics, Hunan Key Laboratory of Tumor Models and Individualized Medicine, The Second Xiangya Hospital, No. 139 Renming Road, Changsha, 410010 Hunan People’s Republic of China

**Keywords:** OS, *LHX9*, FGF, TGF-β, β-Catenin, FRS2

## Abstract

**Background:**

Fibroblast growth factor (FGF) and tumor growth factor-β (TGFβ) have emerged as pivotal regulators during the progression of osteosarcoma (OS). LHX9 is one crucial transcription factor controlled by FGF, however, its function in OS has not been investigated yet.

**Methods:**

The expression of *LHX9*, *FRS2*, *BMP4*, *TGF*-*beta R1*, *SMAD2*, *beta*-*catenin* and metastasis-related proteins was measured by real-time quantitative PCR (RT-qPCR) and Western blot. CCK-8 assay and colony formation assay were employed to determine the proliferation of OS cells, while scratch wound healing assay and transwell assay were used to evaluate their migration and invasion, respectively. In vivo tumor growth and metastasis were determined by subcutaneous or intravenous injection of OS cells into nude mice.

**Results:**

*LHX9* expression was evidently up-regulated in OS tumor tissues and cell lines. Knockdown of *LHX9* impaired the proliferation, migration, invasion and metastasis of OS cells. Mechanistically, *LHX9* silencing led to the down-regulation of BMP-4, β-catenin and metastasis-related proteins, which was also observed in *beta*-*catenin* knockdown OS cells. By contrast, *FRS2* knockdown conduced to the up-regulation of *LHX9*, BMP4, β-catenin and TGF-βR1, while *TGF*-*beta* inhibition repressed the expression of *LHX9* and metastasis-related proteins. Additionally, *let*-*7c* modulates *LHX9* and metastasis-related proteins by suppressing TGF-*beta* R1 expression on transcriptional level.

**Conclusions:**

This study revealed *LHX9* was essential for the proliferation, migration, invasion, and metastasis of OS cells via FGF and TGF-β/β-catenin signaling pathways.

## Background

OS (OS) is one of the most prevalent kinds of malignant bone tumors with high incidence in children and adolescents aged between 15 and 25. The predilection site of OS development is the metaphyseal region of long bones [42]. OS has strong destructive effects on the local bone homeostasis and can easily metastasize to other organs [33], which makes the treatment and recovery more difficult. Current therapy for OS in clinic includes chemo and surgery, however, severe side effects and high recurrence rate restrain the medical output of OS patients. Thus, basic and clinical studies on OS draw extensive attention in orthopedics specialty.

LHX9 belongs to LIM protein family, in which all members contain LIM domain, a specialized double-zinc finger motif that mediates the protein–protein interaction [32]. Previous studies have revealed that there are nine LHX members expressed in mammalian cells, who are named by LIM 1 to 9, and they play essential roles in the development of neuron system [2]. LHX9 has been implicated in the development of brain and heart [35, 46], and the reduced LHX9 expression was associated with the migration and invasion of malignant childhood gliomas [43], however, its function in OS was not clear. Recently, our lab found that the expression of *LHX9* was up-regulated in OS tumor tissues and cells by RNA-seq and RT-qPCR analysis, but the association between OS progression and *LHX9* overexpression was still elusive.

BMP4 is one crucial member of BMP protein family that is part of TGF-β superfamily, which is highly conserved evolutionarily and is essential for Dorsal–Ventral Patterning in development [14]. Like other BMPs, BMP4 is also involved in the bone and cartilage development [38]. Besides BMP4, accumulative evidence shows that Wnt signaling pathway also participates in the regulation of bone development and homeostasis [53]. As one pivotal signal transducer in Wnt pathway, β-catenin can translocate to cell nucleus upon signaling initiation and open the transcription of target genes via associating with transcription factor 4 (TCF4) [30]. Previous studies have found that LHX6 modulated the carcinogenicity of breast cancer cells via β-catenin/TCF4 complex [19], we thus speculated that LHX9 might have the similar regulatory function on OS through β-catenin/TCF4 since it was the homologue of LHX6.

FGF signaling plays a critical role in the development of embryotic organs and tumor progression [4]. Extensive studies have revealed that the engagement of FGF on FGFR could regulate LHX6 and then influenced the expression of downstream BMP4 [54], meanwhile, some reports showed that Wnt/β-catenin could enhance FGF signaling as a positive feedback [31]. FRS2 is the subunit 2 of FGFR, which functions as the bridge between FGF signaling and LMO1. Moreover, bioinformatic analysis demonstrated that LMO1 was modulated by *let*-*7c* and TGF-β receptor (TGF-βR) [44]. Let-7c is one member of let-7 miRNA family, which functions as a tumour suppressor in various cancers [12]. Interestingly, some members of let-7 family, such as let-7a, b, g and i, have been reported to suppress the growth and metastasis of OS cells [28, 49–51]. As the members of LIM family proteins, LHX9 shared the same LIM domain with LMO1, so we speculated that LHX9 might exert similar function like LMO1 in OS development.

In the present study, we focused on the function of LHX9 on regulating OS progression via FGF/FRS2/TGF-β and BMP4/β-catenin signaling. Our study firstly revealed that *LHX9* was up-regulated in OS tissues and cell lines, which was essential for the proliferation, migration, invasion and metastasis of OS cells. Mechanistically, we found that *LHX9* knockdown led to the impairment of metastasis-related proteins, so was for β-catenin knockdown. Nevertheless, *FRS2* silencing elevated the expression of *LHX9* and metastasis-related proteins, which was impaired through TGF-β inhibition by pharmaceutical inhibitors or down-regulation by *let*-*7c*. Our finding uncovered a novel function of LHX9 in the regulation of OS development, the mechanism revealed here would be useful for developing novel small molecular chemicals to treat OS by modulating LHX9 function.

## Results

### OS tissues and cell lines have increased LHX9 expression

**Fig. 1:**
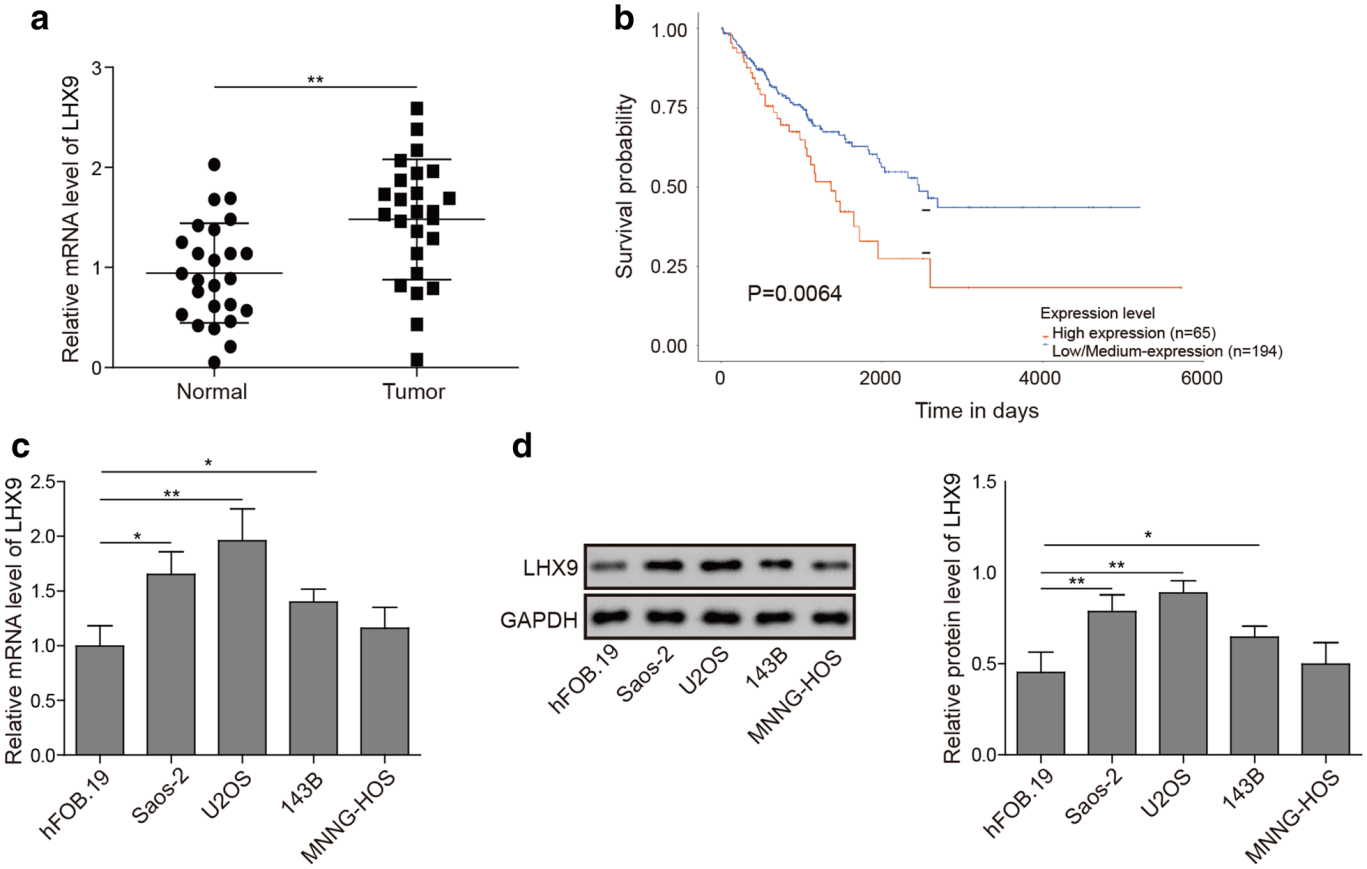
The expression of *LHX9* was up-regulated in OS tissues and cell lines. **a** The relative expression of *LHX9* in normal tissues (solid dots) and OS tumor tissues (solid squares) excised from patients was assessed by RT-qPCR. *LHX9* mRNA levels were normalized to *GAPDH* mRNA (n = 3). **b** The correlation analysis between the expression of *LHX9* and the survival of patients with OS. Blue line: low and medium *LHX9* expression; red line: high *LHX9* expression. **c** The relative expression of *LHX9* in OS cell lines 143B, U2OS, MNNG-HOS and Saos-2 was measured by RT-qPCR. hFOB.19 was the control cell line. The mRNAs were normalized to *GAPDH* mRNA (n = 3). **d** The expression of LHX9 in OS cell lines 143B, U2OS, MNNG-HOS and Saos-2 was determined by Western blot. hFOB.19 was the control cell line, GAPDH was used as the loading control. The relative expression of LHX9 after normalization to GAPDH was shown in the right histogram. **a**–**d** The result was a representative of three independent experiments. **a**, **c**, **d** Error bars represented mean ± SD. *p* values were determined by one-way analysis of variance (ANOVA) followed by Tukey post hoc test (**c**, **d**), unpaired two-tailed Students’ *t*-test (**a**) or log-rank test (**b**). **p < 0.01, *p < 0.05

To comprehensively analyze the differences of gene expression profile between normal cells and OS cells, we firstly collected OS tumor tissues and peritumor tissues from patients and then performed RNA-seq analysis. To our surprise, we noticed that the expression of *LHX9* was much higher in OS tissues when compared with that in normal tissues (data not shown). To see whether other LHX family members had similar expression changes, we further conducted RT-qPCR experiment to measure the relative expression of *LHX1* to *LHX9* (*LHX1*–*9*) with U2OS and hFOB1.19 cells, and the data showed that the expression of *LHX 1*, *7*, *8* was lower, whereas the expression of *LHX2*, *4*, *5*, *6*, *9* was higher in OS tumor tissues, furthermore, *LHX3* expression had no significant change in our experiment (Additional file 1: Figure S1A). Among these genes, *LHX9* had the highest up-regulation, which indicated that *LHX9* might be the most relevant *LHX* member for OS progression. However, we cannot completely rule out the participation of other *LHX* members in OS progression. To confirm this finding, we then performed RT-qPCR experiments with another batch of OS tumor tissues and peritumor tissues from patients, and the data demonstrated that *LHX9* expression was significantly higher in OS tissues when compared with that in normal tissues (Fig. 1a). Then, to explore the potential clinical relevance of *LHX9* in OS progression, we analyzed the survival data of OS patients from TCGA database and found that the survival probability of OS patients with low and moderate *LHX9* expression was significantly higher than those with high *LHX9* expression (Fig. 1b), which hinted that *LHX9* expression might be one hallmark for the prognosis of OS. To confirm the conclusion obtained with patient tumor samples, we further assessed the expression of *LHX9* in various OS cell lines, including 143B, U2OS, MNNG-HOS and Saos-2. As expected, RT-qPCR results displayed that the relative expression of *LHX9* was differently elevated in all these OS cell lines (Fig. 1c). Consistently, Western blot experiment evinced that the expression of LHX9 was indeed higher in various OS cell lines than the control cells, in which U2OS and Saos-2 cells showed the robust increase of LHX9 (Fig. 1d), we thus applied these two cell lines for subsequent experiments. In all, these data revealed that *LHX9* expression was increased in both OS tissues and cell lines, which displayed a negative association with the prognosis of OS.

### *LHX9* knockdown impaired the proliferation of OS cell lines

**Fig. 2:**
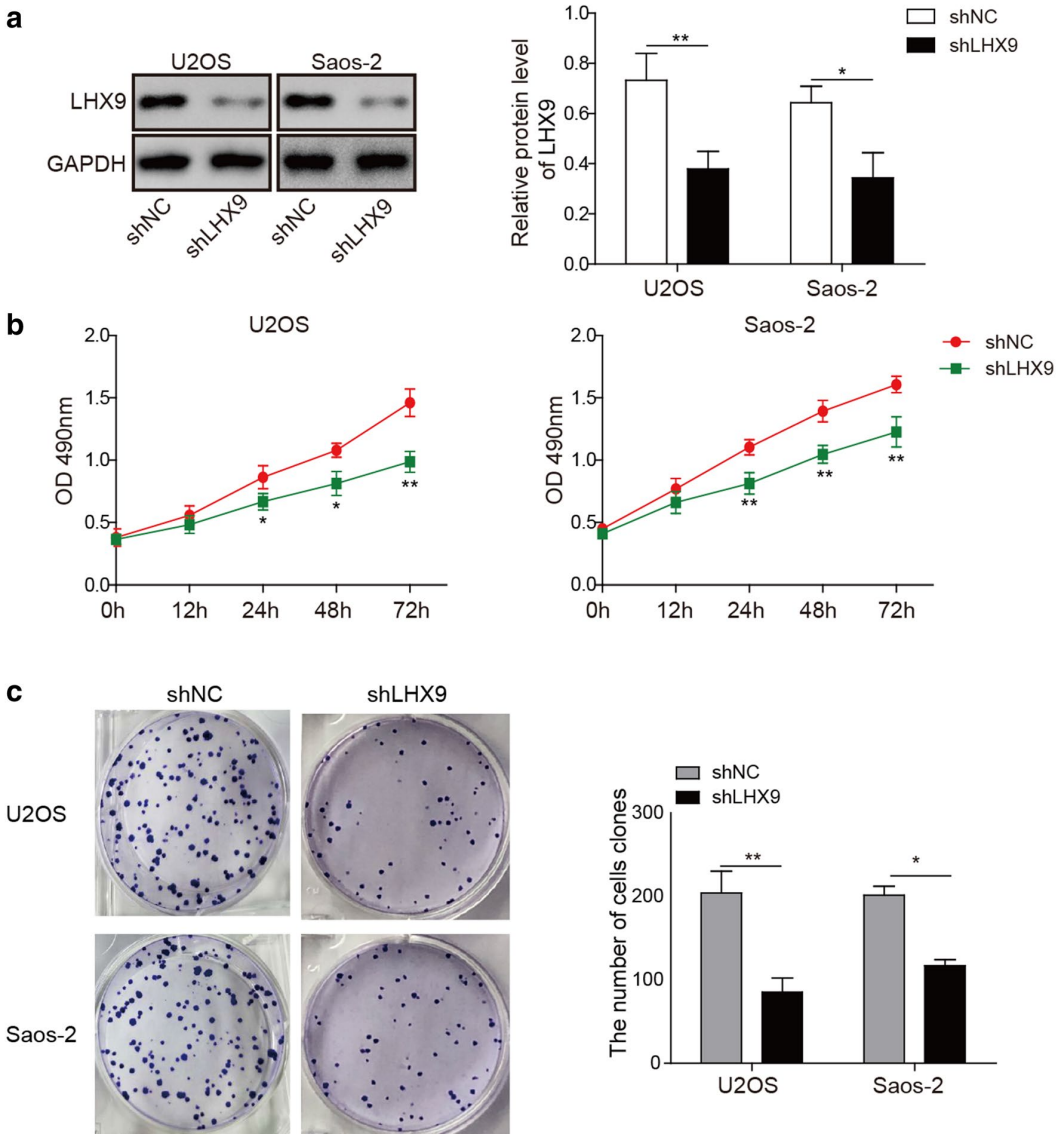
LHX9 knockdown impaired the proliferation of OS cell lines. **a**–**c** U2OS cells or Saos-2 cells were transfected with negative control shRNA (shNC) or shRNA targeting *LHX9* (sh*LHX9*), then they were cultured for 48 h. **a** The expression of LHX9 in shNC- or shLHX9-transfected cells was measured by Western blot. GAPDH was used as the loading control. The relative expression of LHX9 after normalization to GAPDH was shown in the right histogram. **b** The proliferation of shNC- or shLHX9-transfected cells was determined by CCK-8 assay. **c** The survival and proliferation of shNC- or sh*LHX9*-transfected cells were verified by colony formation assay. The statistics of colony formation was shown in the right histograms. **a**–**c** The result was a representative of three independent experiments. **a**–**c** Error bars represented mean ± SD. *p* values were determined by unpaired two-tailed Students’ *t*-test (**a**, **c**) or two-way analysis of variance (ANOVA) with Tukey’s post hoc test (**b**). **p < 0.01, *p < 0.05

To investigate the role of LHX9 in the development of OS, we firstly knocked down *LHX9* in U2OS and Saos-2 cells using shRNAs. Western bolt results showed that the transfection of sh*LHX9* plasmid led to obvious down-regulation of LHX9 in both U2OS and Saos-2 cells, and the knockdown efficiency was about 50% (Additional file 1: Figure S1B), which indicated that shRNA-mediated *LHX9* knockdown was successful (Fig. 2a). Next, we analyzed the effect of *LHX9* knockdown on the proliferation of OS cells by CCK-8 assay, as expected, the data demonstrated that knockdown of LHX9 significantly impaired the proliferation of U2OS and Saos-2 cells (Fig. 2b). Furthermore, we performed colony formation assay to assess the proliferation capacity of shNC- and shLHX9-transfected OS cells, in consistent with the CCK-8 assay, colony formation results demonstrated that *LHX9* knockdown severely repressed the proliferation and colony formation of OS cells (Fig. 2c). Taken together, our data elucidated that LHX9 was essential for the proliferation of OS cells.

### The migration, invasion and metastasis of OS cell lines were attenuated by *LHX9* knockdown

**Fig. 3:**
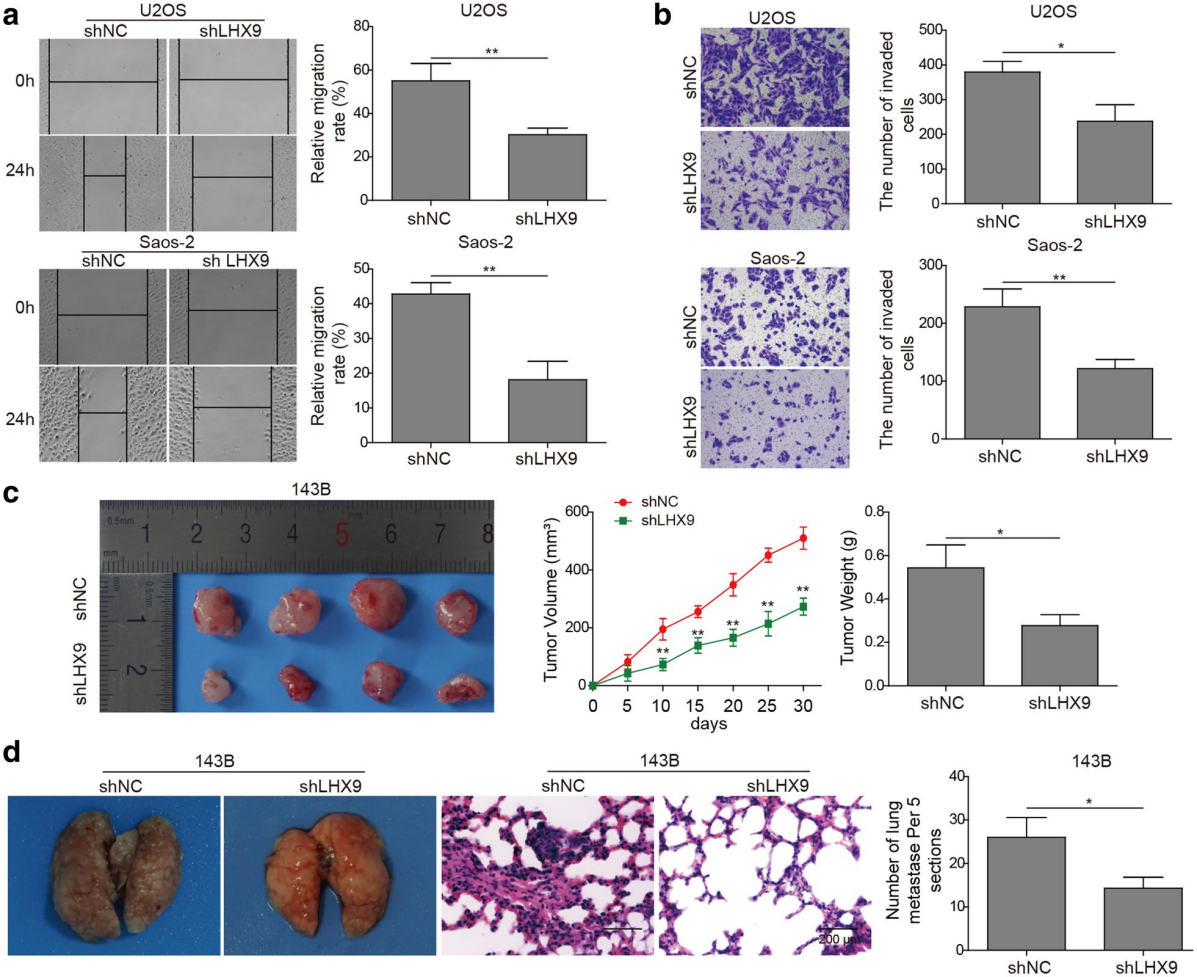
The migration, invasion and metastasis of OS cell lines were attenuated by *LHX9* knockdown. **a**–**d** U2OSor Saos-2 cells were transfected with shNC or sh*LHX9*, then they were cultured for 48 h. **a** The migration of shNC- or sh*LHX9*-transfected cells was measured by scratch wound healing assay. The statistics of wound healing rate was shown in the right histograms. **b** The invasion of shNC- or sh*LHX9*-transfected cells was determined by transwell assay. The statistics of cells invaded to the lower chamber was shown in the right histograms. **c** shNC- or sh*LHX9*-transfected 143B cells were transplanted to 6-week-old nude mice via subcutaneous injection (n = 10). The tumor growth was monitored by measuring the tumor volume every 5 days. 30 days later, the tumor tissues were obtained from euthanized mice, imaged and weighted. The statistics of tumor weights was shown in the right histograms. **d** shNC- or sh*LHX9*-transfected 143B cells were intravenously injected into 6-week-old nude mice (n = 10). 36 days later, mice were euthanized, and the tumor multiplicity on lungs was imaged. Fixed lung tissues were then sectioned for H&E staining, the metastatic intrapulmonary nodules were imaged and counted. The statistics of metastatic nodules in lungs (five sections per lung) was shown in the right histograms. **a**–**d** The result was a representative of three independent experiments. Error bars represented mean ± SD. *p* values were determined by unpaired two-tail Students’ *t*-test [**a**, **b**, **c** (tumor weight data), **d**] or two-way analysis of variance (ANOVA) followed by Tukey post hoc test (**c**, tumor volume data). **p < 0.01, *p < 0.05

OS was one kind of bone tumor which could metastasize to lungs. To verify the effect of LHX9 on the migration, invasion and metastasis of OS cells, we firstly performed scratch wound healing assay with shNC- or shLHX9-transfected U2OS and Saos-2 cells. As shown in Fig. 3a, *LHX9* knockdown led to much slower wound healing rate and impaired cell migration capacity when compared with that in shNC-transfected OS cells (Fig. 3a). Moreover, transwell assay was employed to evaluate the cell invasion capacity of OS cells in the absence or presence of *LHX9*. Consistently, both U2OS and Saos-2 cells displayed attenuated cell invasion if *LHX9* was knocked down (Fig. 3b). Then, we aimed to figure out the influence of *LHX9* knockdown on the growth and metastasis of OS cells in vivo. shNC- or sh*LHX9*-transfected 143B cells were transplanted into nude mice via subcutaneous injection, and the tumor growth was monitored by measuring the tumor volume every week. We observed a much slower tumor growth for *LHX9*-knockdown OS cells. Meanwhile, the tumor volume was smaller in nude mice transplanted with *LHX9*-knockdown OS cells after 4-weeks, which implicated that the growth of OS cells was dampened after *LHX9* knockdown (Fig. 3c). Lastly, we performed tumor metastasis assay by intravenously injection of shNC- or sh*LHX9*-transfected 143B cells into nude mice, which was implemented to ascertain the effect of LHX9 on tumor cell metastasis. In accord with the in vitro data, metastatic tumor multiplicity on lung tissues derived from *LHX9*-knockdown cells was evidently lower than that derived from shNC-transfected cells (Fig. 3d), which meant that the metastasis of OS cells was inhibited if *LHX9* was lacking. In summary, our data here suggested that the LHX9 was required for the migration, invasion and metastasis of OS cells, while its knockdown reduced these capabilities of OS cells.

### *LHX9* knockdown led to the down-regulation of BMP-4, β-catenin and metastasis-related proteins

**Fig. 4:**
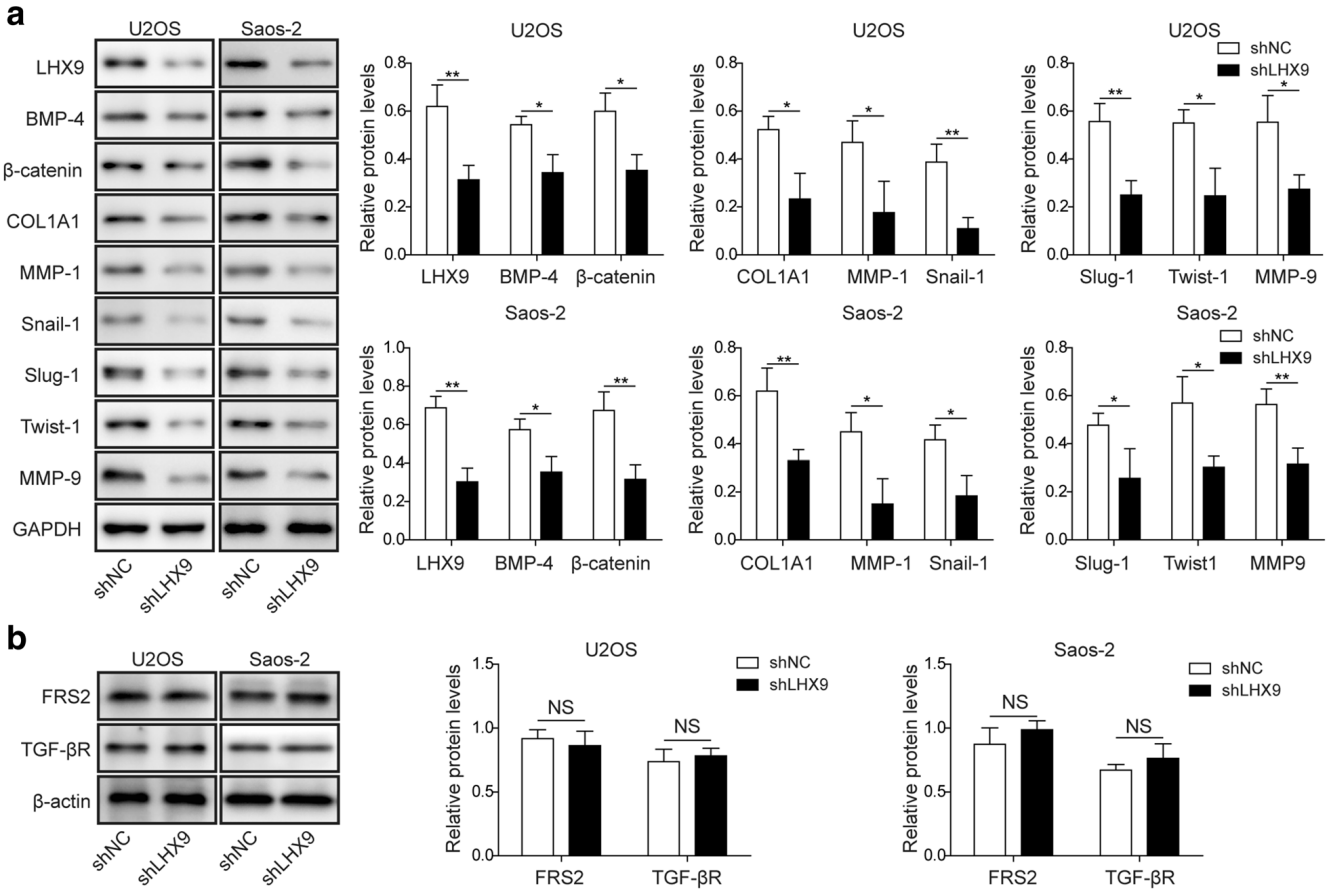
*LHX9* knockdown led to the down-regulation of BMP-4, β-catenin, COL1A1, MMP-1, Snail-1, Slug-1, Twist1 and MMP-9. **a**, **b** U2OS or Saos-2 cells were transfected with shNC or sh*LHX9*, then they were cultured for 48 h. The expression of LHX9, BMP-4, β-catenin, COL1A1, MMP-1, Snail-1, Slug-1, Twist-1, MMP-9 (**a**) and FRS2, TGF-βR1 (**b**) was measured by Western blot. GAPDH was used as the loading control. The normalized expression of these proteins against GAPDH was shown in the right histograms. The result was a representative of three independent experiments. Error bars represented mean ± SD. *p* values were determined by unpaired two-tail Students’ *t*-test. **p < 0.01, *p < 0.05*. NS* not significant

Previous study found that LHX2 and LHX4 could modulate the carcinogenicity and metastasis of various tumors through β-catenin/TCF4 pathway [7, 24], so we wonder whether the homologous LHX9 had similar function in OS. To address this question, we then perform western blot to probe the expression of related proteins in BMP4 and Wnt signaling pathways, such as BMP4, β-catenin, COL1A1, Snail-1, Slug-1, MMP-1, Twist1 and MMP-9, all of which were involved in the metastasis process [16–18, 26, 29, 47]. As expected, *LHX9* knockdown led to reduced expression levels of all these proteins (Fig. 4). Taken together, our data elucidated that LHX9 was indispensable to maintain the homeostasis of BMP4 and Wnt signaling pathways that controlled the metastasis process.

### *Beta*-*catenin* knockdown reduced the expression of LHX9 and metastasis-related proteins

**Fig. 5:**
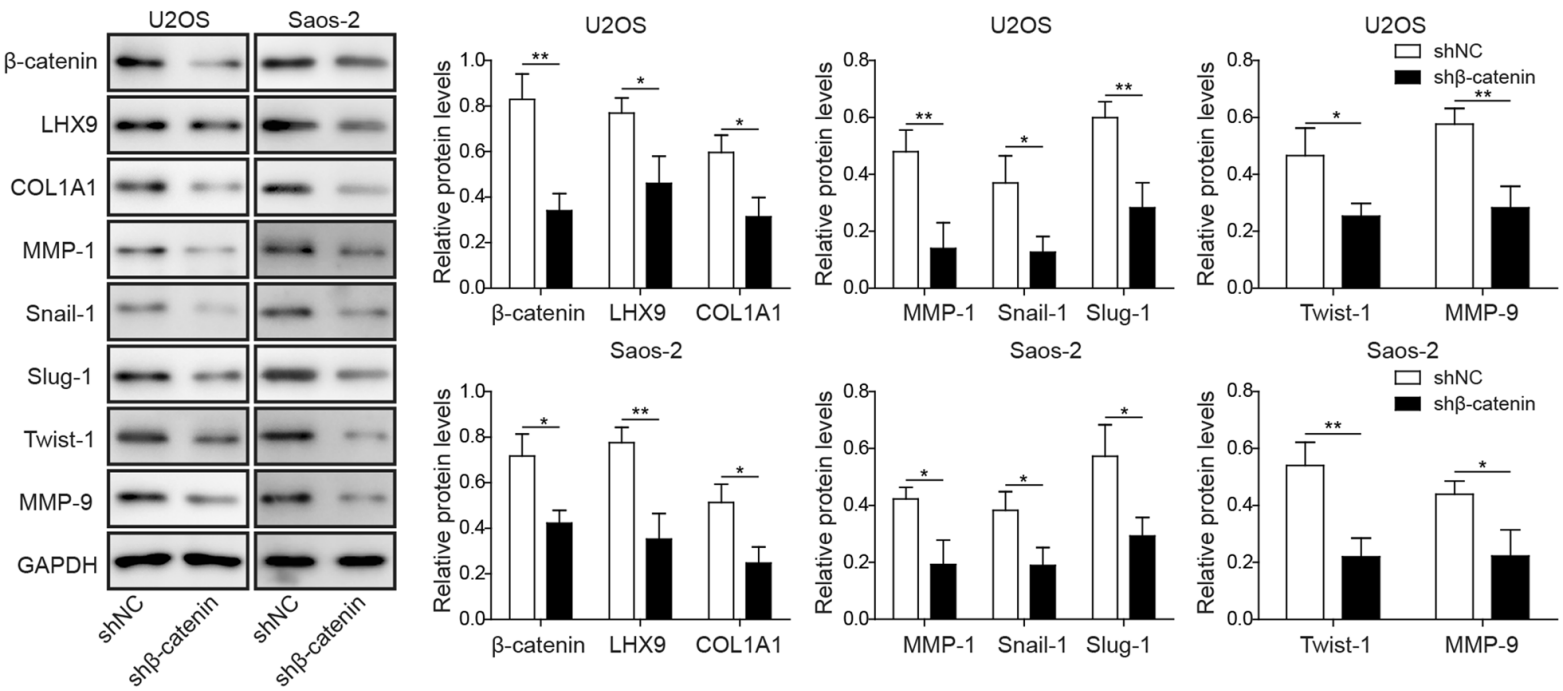
*Beta*-*catenin* knockdown resulted in the attenuation of LHX9 and metastasis-related proteins. U2OS or Saos-2 cells were transfected with shNC or shRNA targeting *beta*-*catenin* (sh*beta*-*catenin*), then they were cultured for 48 h. The expression of β-catenin, LHX9, COL1A1, MMP-1, Snail-1, Slug-1, Twist-1 and MMP-9 was assessed by Western blot. GAPDH was used as the loading control. The normalized expression of these proteins against GAPDH was shown in the right histograms. The result was a representative of three independent experiments. Error bars represented mean ± SD. *p* values were determined by unpaired two-tail Students’ *t*-test. **p < 0.01, *p < 0.05

To investigate the role of β-catenin in the regulation of metastasis and the relationship between LHX9 and β-catenin, we next knocked down beta-catenin in U2OS and Saos-2 cells, and then assessed the expression of LHX9, β-catenin, COL1A1, Snail-1, Slug-1, MMP-1, Twist1 and MMP-9, respectively. The Western blot experiment demonstrated the knockdown efficiency of β-catenin was more than 50%, suggesting that the shRNA-mediated β-catenin knockdown was successful (Additional file 1: Figure S1B). Consistent with the results we obtained from LHX9 knockdown cells, β-catenin-knockdown in OS cells also led to the attenuation of all these proteins, which was evinced by Western blot experiments (Fig. 5). These data revealed that Wnt/β-catenin pathway was essential for the metastasis of OS cells, and β-catenin could be regulated by LHX9 in a positive feedback.

### FRS2 negatively regulated LHX9 via restraining TGF-βR1 and BMP-4 expression

**Fig. 6:**
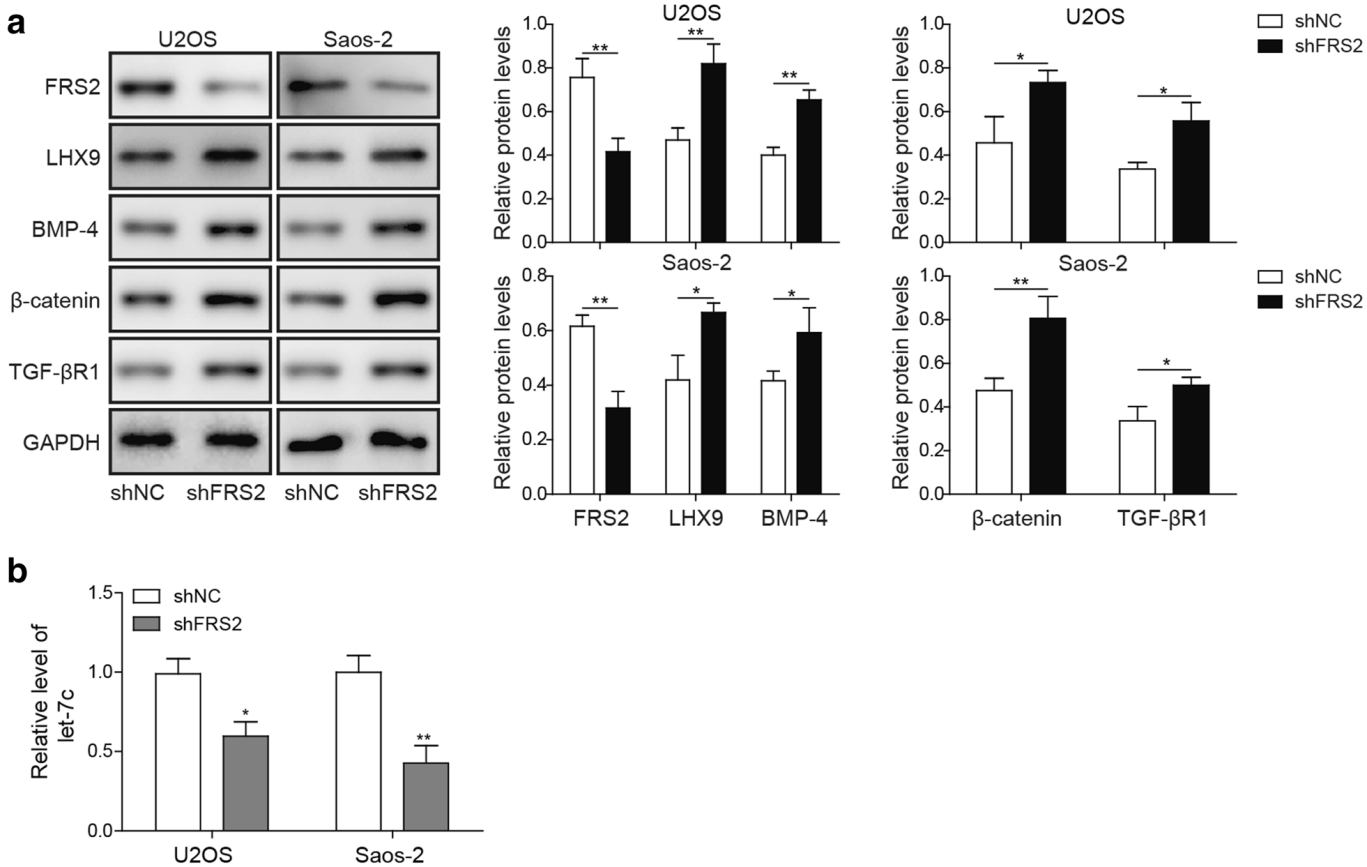
*FRS2* knockdown resulted in the up-regulation of LHX9, BMP4, β-catenin, *let*-*7c* and TGF-βR1. **a**, **b** U2OS or Saos-2 cells were transfected with shNC or shRNA targeting *FRS2* (sh*FRS2*), then they were cultured for 48 h. **a** The expression of LHX9, BMP4, β-catenin, and TGF-βR1 was assessed by Western blot. GAPDH was used as the loading control. The normalized expression of these proteins against GAPDH was shown in the right histograms. **b** The expression of *let*-*7c* was measured by RT-qPCR. *Let*-*7c* were normalized to *GAPDH mRNA* (n = 3). **a**, **b** The result was a representative of three independent experiments. Error bars represented mean ± SD. *p* values were determined by unpaired two-tail Students’ *t*-test (**a**, **b**). **p < 0.01, *p < 0.05

Previous study found that FGF signaling regulated LHX9 and its downstream protein BMP4 [36], as one crucial adaptor protein at the downstream of FGFR, FRS2 has been reported to mediate the association between FGF and LMO1, which was also modulated by *let*-*7c* and TGF-βR1 [44]. Since LHX9 shared the same domain with LMO1, we thus speculated that LHX9 might be regulated by FGF signaling and *let*-*7c* plus TGF-βR1. To verify this hypothesis, we used shRNA to knock down *FRS2* in U2OS and Saos-2 cells, and the Western blot experiment demonstrated the knockdown efficiency of FRS2 was about 50%, suggesting that the shRNA-mediated FRS2 knockdown was successful (Additional file 1: Figure S1B). We then detected the expression of *LHX9, BMP*-*4, beta*-*catenin*, *let*-*7c* and *TGF*-*beta R1* with RT-qPCR and Western blot, respectively. As expected, *FRS2* knockdown led to the augmentation of *LHX9, BMP*-*4, beta*-*catenin*, *TGF*-*beta R1* and the impairment of *let*-*7c* on transcription level, moreover, biomedical evidence confirmed the up-regulation of LHX9, BMP-4, β-catenin, TGF-βR1 in *FRS2*-knockdown cells (Fig. 6a, b). Here, our experiments demonstrated that the down-regulation of FRS2 would boost the expression of *LHX9, BMP*-*4, beta*-*catenin* and *TGF*-*beta R1* in FGF, Wnt/β-catenin and TGF-βR1 pathways.

### TGF-βR1 signaling was involved in the regulation of LHX9 and downstream proteins

**Fig. 7:**
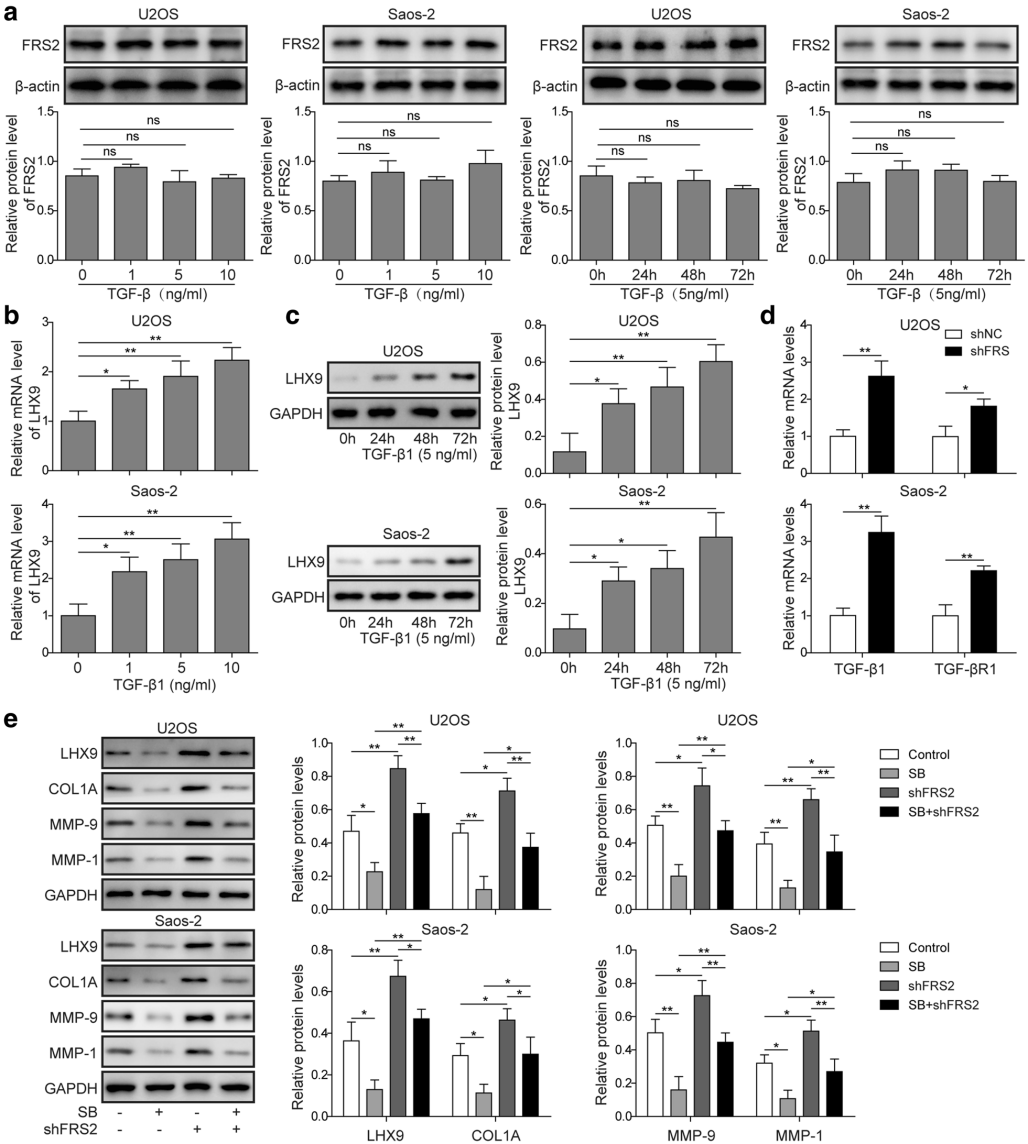
TGF-β signaling was involved in the regulation of LHX9 and metastasis-related proteins. **a** U2OS or Saos-2 cells were cultured without or with 1, 5, 10 ng/ml TGF-β1 for 24 h (left two panels), or cultured without or with 5 ng/ml TGF-β1 for 24, 48 and 72 h, respectively (right two panels). The expression of FRS2 protein was measured by Western blot, β-actin was used as the loading control. The normalized protein expression levels against β-actin were shown in the below histograms. **b** U2OS or Saos-2 cells were cultured without or with 1, 5, 10 ng/ml TGF-β1 for 24 h, then the relative expression of *LHX9* was assessed by RT-qPCR. The mRNAs were normalized to *GAPDH*, experiments were performed in triple. **c** U2OS or Saos-2 cells were cultured without or with 5 ng/ml TGF-β1 for 24, 48 and 72 h, respectively, and the relative expression of LHX9 was measured by Western blot. GAPDH was used as the loading control. The normalized expression of LHX9 against GAPDH was shown in the right histograms. **d** U2OS or Saos-2 cells were transfected with shNC or sh*FRS2*, then they were cultured for 48 h. The relative expression of TGF-*beta* and TGF-*beta* R1 was assessed by RT-qPCR. The mRNAs were normalized to *GAPDH* mRNA (n = 3). **e** U2OS or Saos-2 cells were transfected with shNC or sh*FRS2*, then they were cultured in the absence or presence of TGFβR1 inhibitor (SB431542) for 48 h. The expression of LHX9, COL1A1, MMP-9, and MMP-1 was measured by Western blot. GAPDH was used as the loading control. The normalized expression of these proteins against GAPDH was shown in the right histograms. **a**–**e** The result was a representative of three independent experiments. Error bars represented mean ± SD. *p* values were determined by one-way analysis of variance (ANOVA) followed by Tukey post hoc test (**a**–**c**, **e**) or unpaired two-tail Students’ *t*-test (**d**). **p < 0.01, *p < 0.05. *NS* not significant

To further validate the effect of TGF-βR1 signaling on *LHX9* expression, we firstly applied different doses of TGF-β1 to treat U2OS and Saos-2 cells and then measured the relative expression of *LHX9* via RT-qPCR. The result showed that *LHX9* expression was enhanced after TGF-β1 stimulation, which also displayed a dose-dependent manner (Fig. 7a). At the same time, we cultured U2OS and Saos-2 cells with a constant dose of TGF-β1 for different time, subsequent Western blot experiment evinced that LHX9 expression was up-regulated with a time-dependent feature (Fig. 7b). In previous experiments, we have identified that *FRS2* knockdown led to the up-regulation of both LHX9 and TGF-βR1, herein, we further found that *FRS2* knockdown induced the up-regulation of TGF-β1 in U2OS cells (Fig. 7c). Moreover, we administrated control shRNA- or sh*FRS2*-transfected U2OS and Saos-2 cells with TGF-βR1 inhibitor SB431542, in keeping with previous results, *FRS2* knockdown led to the elevation of LHX9, COL1A1, MMP-9 and MMP-1 in Western blot experiment, which meant that the metastasis was enhanced. However, TGF-βR1 inhibition robustly repressed the expression of these proteins (Fig. 7d), indicating that the metastasis process was impaired. In all, our data revealed that TGF-βR1 signaling positively regulated the expression of LHX9 and its downstream proteins, suggesting that it was essential for LHX9 mediated metastasis.

### The regulation of LHX9 and metastasis-related proteins by TGF-βR1 was influenced by *let*-*7c*

**Fig. 8:**
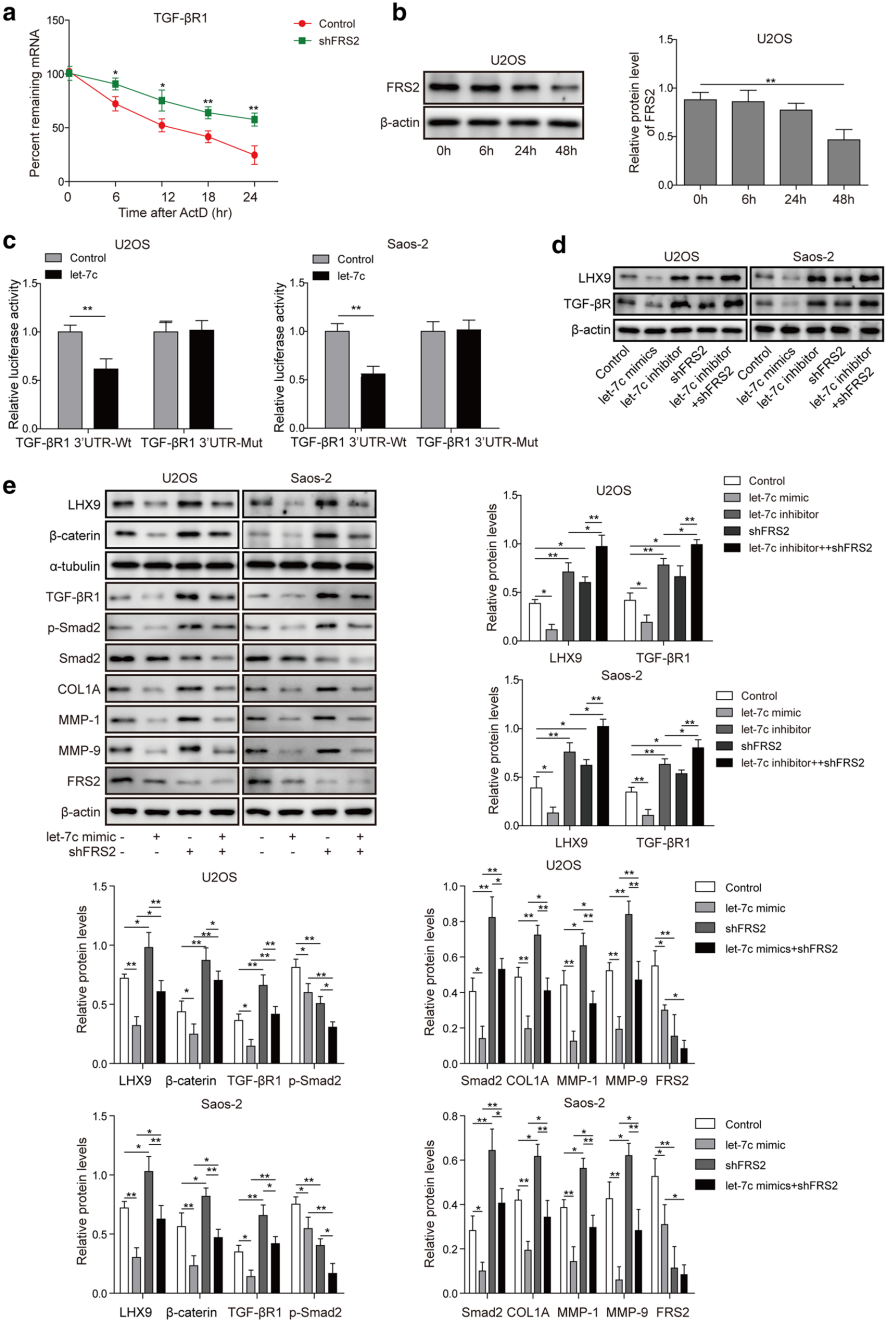
*Let*-*7c* participated in the regulation of LHX9 and metastasis-related proteins by repressing TGF-*beta* R1 on transcriptional level. **a** U2OS cells were transfected with shNC or sh*FRS2*, then they were cultured in the absence or presence of transcription inhibitor (ActD) for 24 h. The relative expression of *TGF*-*beta R1* was assessed by RT-qPCR every 6 h during the culturing time. The *TGF*-*beta R1* mRNAs were normalized to *GAPDH mRNA* (n = 3). **b** U2OS cells were transfected with sh*FRS2*, then they were cultured for 0 h, 6 h, 24 h and 48 h, respectively. The expression of FRS2 protein was assessed by Western blot, β-actin was used as the loading control. The normalized protein expression levels against β-actin were shown in the right histograms. **c** U2OS or Saos-2 cells were co-transfected with *let*-*7c* mimic control/*let*-*7c* mimic and Wt-TGF-βR1 3′UTR/Mut-TGF-βR1 3′UTR. Dual luciferase assay was performed to measure the luciferase activity. **d** U2OS or Saos-2 cells were un-transfected or transfected with sh*FRS2*, then they were cultured without or with *let*-*7c* mimic or inhibitor for 48 h, then the expression of LHX9 and TGF-βR1 was assessed by Western blot. β-actin was used as the loading control. The normalized protein expression levels against β-actin were shown in the below histograms. **e** U2OS or Saos-2 cells were transfected with shNC or sh*FRS2*, then they were cultured in the absence or presence of *let*-*7c* mimic for 48 h. The expression of LHX9, beta-catenin, TGF-βR1, p-Smad2, Smad2, COL1A1, MMP-9, MMP-1 and FRS2 was measured by Western blot. β-actin was used as the loading control. The normalized protein expression levels against β-actin were shown in the below histograms. **a**–**e** The result was a representative of three independent experiments. Error bars represented mean ± SD. *p* values were determined by two-way analysis of variance (ANOVA) (**a**) or one-way analysis of variance (ANOVA) followed by Tukey post hoc test (**b**–**e**). **p < 0.01, *p < 0.05, *ns* not significant

To explore the underlying mechanism for the regulation of LHX9 by TGF-βR1 signaling, we then treated control shRNA- or sh*FRS2*-transfected U2OS cells with transcription inhibitor-Actinomycin D (ActD) for different time, the subsequent RT-qPCR result exhibited that *TGF*-*beta R1* mRNA continued to decline with time (Fig. 8a), indicating that other players participated in the down-regulation of *TGF*-*beta R1* mRNA on transcriptional level. At the same time, Western blot results showed that the knockdown of FRS2 protein was not so obvious at 24 h after the transduction of shRNA, whereas the knockdown efficiency reached to about 50% at 48 h post the transduction (Fig. 8b). Previous study has revealed that *let*-*7c* could target *TGF*-*beta R1* mRNA on two seeding sites. Next, we designed and performed dual luciferase assay to confirm this interaction, and the results illustrated that *let*-*7c* co-transfection suppressed the expression and activity of luciferase (Fig. 8c), which indicated that *let*-*7c* could interact with *TGF*-*beta R1* mRNA to repress its translation. To confirm the inhibitory effect of *let*-*7c* on TGF-βR1, we then transfected U2OS cells and Saos-2 cells with *let*-*7c* mimic or inhibitor, respectively, then the expression of LHX9 and TGF-βR1 was assessed by Western blot. As expect, the results displayed that let-7c mimic significantly attenuated the expression of LHX9 and TGF-βR1, while let-7c inhibitor potently enhanced their expression. Moreover, FRS2 knockdown also led to the up-regulation of both LHX9 and TGF-βR1, and additional let-7c inhibitor treatment further enhanced their expression levels (Fig. 8d). Moreover, we further assessed the expression of representative proteins in TGF-β1 pathway and key proteins involved in metastasis, such as TGF-βR1, p-Smad2, Smad2, COL1A1, MMP-9, MMP-1, we well as LHX9 and beta-catenin, upon FRS2 knocking down and *let*-*7c* overexpression. It was noted that excessive *let*-*7c* in U2OS and Saos-2 cells led to the down-regulation of proteins mentioned above, while FRS2 knockdown resulted in the elevation of them except for Smad2, however, the combination of *let*-*7c* overexpression and *FRS2* knockdown severely repressed their expression (Fig. 8e). In summary, these experiments suggested that TGF-βR1 participated in the regulation of LHX9 via *let*-*7c*, which was closely associated with the metastasis of OS cells.

### LHX9 regulates OS development through interacting withβ-catenin and promoting its nuclear translocation

**Fig. 9:**
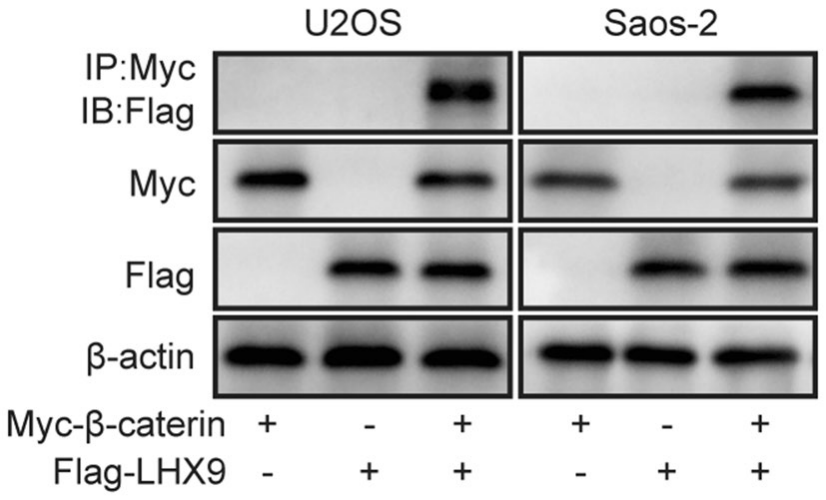
LHX9 regulates OS development through binding withβ-catenin and inhibiting its degradation. **a** U2OS cells or Saos-2 cells were transfected with Myc-tagged beta-catenin, Flag-tagged LHX9 or both, then they were cultured for 48 h. The interaction between beta-catenin and LHX9 was assessed by Co-IP experiment. β-actin was used as the loading control

The above evidence proved that the regulation of OS progression and metastasis by LHX9 is beta-catenin dependent, however, to further reveal the detailed association between LHX9 and beta-catenin, we then overexpressed Myc-tagged beta-catenin and Flag-tagged LHX9 in U2OS cells and Saos-2 cells, respectively. Afterwards, we performed co-immunoprecipitation assay and the data elucidated that LHX9 could interact with beta-catenin (Fig. 9), similarly, LHX4 has also been reported to bind to beta-catenin, which then translocated into cell nuclear and facilitated the association of TCF4 with LHX4/beta-catenin complex, and the complex could transactive downstream genes involved in the progression of human colorectal cancer [7]. Based on our experiments and previous reports, we speculated that LHX9 promoted the nuclear translocation of beta-catenin through their interaction, which then facilitated gene expression that were controlled by beta-catenin and boosted OS progression and metastasis.

## Discussion

Emerging evidence has showed that LHX9 was necessary for the development of many organs in embryos, including gonads, limbs, heart and the nervous system [3, 27, 41, 48]. However, little was known about its role in tumor initiation and progression. In present study, we used various assays to identify the novel function of LHX9 on promoting metastasis of OS cells via BMP-4 and β-catenin pathways. At first, we found that the expression level of *LHX9* was significantly up-regulated in OS tumor tissues and cell lines. To investigate its function, we then knocked down *LHX9* in U2OS or Saos-2 cells, the following CCK-8 and colony formation assays demonstrated that cell proliferation was impaired. Moreover, the migration and invasion capacity of OS cells were dampened after *LHX9* knockdown. At last, we compared the tumor growth and metastasis of OS to lung with or without *LHX9* in vivo, the data also supported that *LHX9* was required for the growth and metastasis of OS. To our knowledge, this is the first report about the regulation of OS by *LHX9*. In addition, *LHX9* expression was found to be reduced during the development of gliomas in children, which was correlated with the migration and invasion of glioma [43]. As for other LHX members, early studies revealed that LHX2 promoted tumor cell proliferation in pancreatic ductal adenocarcinoma, and LHX4 facilitated the development of colorectal cancer, both via enhancing Wnt/TCF4/β-catenin pathway [7, 55]. Therefore, the function of LHXs may vary in different tumors and in different status of tumor development.

TGF-β-Smad2 signaling pathway played an essential role in the carcinogenesis [13]. In previous studies, it has been found that enhanced TGF-beta expression and strengthened TGF-β signaling resulted in the augmented phosphorylation of Smad2 [21]. In this study, we revealed that LHX9-mediated tumorigenesis depended on TGF-β signaling, evinced by the up-regulation of *LHX9* upon excessive TGF-β stimulation and the down-regulation of *LHX9* after TGF-βR inhibition or knockdown. Mechanistically, we speculated that Smad2 might mediate the effect of TGF-β signaling on LHX9, however, we did not have direct evidence to demonstrate the association between Smad2 phosphorylation and *LHX* expression, which was not studied in previous reports yet. Thus, investigating the detailed regulatory mechanism between TGF-β and LHX9 was one important work in the following study.

FRS2 was one crucial docking protein in FGF signaling cascade, which also participated in the regulation of TGF-β signaling [39]. In our study, we found that knockdown of FRS2 resulted in the augmentation of TGFβ signaling and the up-regulation of *LHX9* as well as its downstream proteins, including BMP-4 and β-catenin. The association between FRS2 and TGF-β has been verified in previous studies that *let*-*7* miRNA mediated the regulation of FGF on TGF-βR1 [9]. Through bioinformatic prediction and dual-luciferase assay, we confirmed that *let*-*7c* specifically bound to 3′-UTR of *TGF*-*beta R1* mRNA on two different sites, which mediated its degradation and thus suppressed the expression of *TGF*-*beta R1*. Moreover, the knockdown of *FRS2* led to the attenuation of *let*-*7c*, which further conduced to the elevation of *TGF*-*beta R1* and *LHX9*. Our results were consistent with previous reports that FGF signaling was required for *LHX9* expression in the development of brain, eyes and limb [1, 10, 48]. In addition, other LHX members, such as LHX2, mediated the FGF-Sonic hedgehog (SHH) regulatory loop in limb development [45]; *LHX8* expression was regulated by retinoic acid through FGF-8b in the upper jaw development of chicken embryo [40]; *LHX6* and *LHX7* was regulated by different FGF ligands during the branchial arch and tooth development in zebrafish [20]. In all, our data further confirmed that FGF-TGF-β signaling crosstalk was the key regulator of *LHX9* expression and function in the proliferation, migration and metastasis of OS cells.

Besides of TGFβ signaling, previous studies have revealed that several highly conserved signaling pathways are also associated with the progression and metastasis of OS. For example, hedgehog signaling (Hh) pathway, receptor activator for nuclear factor-κB (RANK)/RANKL pathway, Wnt pathway, Notch pathway, phosphatidylinositol 3-kinase (PI3K)/Akt/mammalian target of rapamycin (mTOR) pathway. Accumulative evidence suggests that aberrant Hh signaling is observed in both OS cell lines and human OS specimens, which promotes the proliferation and migration of OS cells, and its inhibitors could reduce the proliferation and growth of OS [23]. RANK/RANKL signaling is essential for bone homeostasis and is implicated in OS development [34], and the combination of RANKL inhibitor denosumab with sorafenib leads to complete metabolic remission of an OS patient in one clinical study [6]. Canonical Wnt/β-catenin/TCF/lymphoid enhancer factor pathway is crucial for bone morphogenesis, bone mass regulation and bone regeneration [5], and the enhanced Wnt signaling has been confirmed in primary human OS tissues and OS cell lines [8]. Various Wnt antagonists have been tested in in vitro and in vivo experiments and exhibit down-regulated proliferation, migration and metastasis of OS [25, 37]. Notch and PI3K/Akt/mTOR also contribute to the development of OS, and the inhibitors targeting PI3K, Akt, mTOR or Notch signaling demonstrates robust suppression on the proliferation of OS cells in vitro and the OS tumor growth in vivo [15, 22, 52]. Based on the above evidence, we speculate that targeting TGFβ signaling might be another approach to repress the proliferation, migration and metastasis of OS cells.

At last, we observed that LHX9 promoted the expression of β-catenin through suppressing GSK3β, which then led to the enhancement of β-catenin. Meanwhile, the knocking down experiments also confirmed the positive correlation between LHX9 and β-catenin. Mechanistic study further revealed that LHX9 could interact with β-catenin, which might help to stabilize β-catenin and prevent its degradation. As one pivotal transcription factor, β-catenin could bind to the promoter region of target genes with TCF and then initiate their expression. Moreover, β-catenin could further enhance the TGFβ-Smad2 signaling as a positive feedback [11], while its silencing significantly impaired the expression of *LHX9* and metastasis-related proteins. Thus, targeting β-catenin may represent one feasible therapy for OS in future.

## Conclusions

Although we have revealed a novel function of LHX9 in the growth and metastasis of OS via in vitro and in vivo methods, there were still some questions needed to be addressed, especially for the detailed regulatory mechanism in FRS2-TGF-β-Smad2-LHX9-β-catenin axis. Next, we plan to confirm our conclusions by performing routine assays with LHX9 overexpressed cells.

## Methods

### Reagents

ATCC-formulated McCoy’s 5a Medium Modified, Eagle’s Minimum Essential Medium (EMEM), and Leibovitz’s L-15 Medium were purchased from American Type Cell Culture (ATCC, Manassas, VA, USA). Ham’s F12 Medium, Dulbecco’s Modified Eagle’s Medium (DMEM), l-glutamine, Geneticin (G418), Lipofectamine 3000, TRIzol Plus RNA Purification Kit, SuperScript IV First-Strand Synthesis System, Pierce ECL western blotting substrate were obtained from Thermo Fisher Scientific (Carlsbad, CA, USA). Rabbit anti-human LHX9 antibody (ab224357), rabbit anti-human BMP4 antibody (ab39973), rabbit anti-human β-catenin antibody (ab32572), rabbit anti-human COL1A antibody (ab34710), anti-MMP-1 antibody (1:1000, Abcam-ab137332), anti-Snail-1 antibody (1:1000, Abcam-ab53519), anti-Slug-1 antibody (1:1000, Abcam-ab106077), rabbit anti-human Twist1 antibody (ab49254), rabbit anti-human MMP-9 antibody (ab73734), rabbit anti-human TGF-β receptor 1 antibody (ab31013) were from Abcam (Cambridge, UK). Mouse anti-human FRS2 antibody (462910) was bought from Novus Biologicals. Mouse anti-human Smad2 (L16D3) antibody, mouse anti-Myc tag (9B11) antibody, rabbit anti-human phospho-Smad2 (Ser465/467) (138D4) antibody, rabbit anti-GAPDH (D16H11) XP antibody and rabbit anti-β-actin (13E5) antibody was from Cell Signaling Technology (Danvers, MA, USA). Mouse anti-Flag tag antibody was from Sigma-Aldrich (Munich, Germany), Goat anti-mouse IgG-HRP and goat anti-rabbit IgG-HRP antibodies were from Santa Cruz (Dallas, TX, USA). All the primers, *let*-*7c* mimic and inhibitor, hematoxylin–eosin (HE) staining kit, Cell Counting kit-8 (CCK-8) kit, BCA protein assay kit were synthesized or bought in Shanghai Sangon Biotech (Shanghai, China). SYBR Green qPCR Master Mix was obtained from Bio-Rad (Hercules, California, USA). pmirGLO Dual-Luciferase miRNA Target Expression Vector and Dual-Glo Luciferase Assay System were from Promega (Madison, WI, USA). The transwell system was bought from Corning (Tewksbury, MA, USA). SB431542 and Actinomycin D (ActD) was obtained from Sigma-Aldrich (Munich, Germany). pLKO.1, psPAX2 and pMD2.G plasmids were obtained from Addgene.

### Clinical studies

OS and peritumoral tissues were obtained from 25 patients via surgical excision, which was approved by the Ethics Review Committee of the Second Xiangya Hospital of Central South University. Written informed consent were all signed by the enrolled patients. All the samples were immediately frozen in liquid nitrogen and stored in − 80 °C fridge until RNA extraction.

### TCGA data collection

Expression data of LHX9 was obtained from the publicly available TCGA datasets which were directly downloaded from the TCGA Data Portal at https://tcga-data.nci.nih.gov/tcga/. Gene expression data were available for 65 in LHX9-high groups, and 194 in LHX9-median plus low groups in OS samples.

### Cell culture and treatment

Human OS cell lines 143B, U2OS, MNNG-HOS Saos-2 and hFOB 1.19 were purchased from American Type Cell Culture (ATCC, Manassas, VA, USA). U2OS, 143B and Saos-2 cells were cultured in ATCC-formulated McCoy’s 5a Medium Modified supplemented with 10% fetal bovine serum (FBS) and 1% penicillin and streptomycin. MNNG-HOS cells were cultured in ATCC-formulated Leibovitz’s L-15 Medium supplemented with 10% FBS and 1% penicillin and streptomycin. hFOB 1.19 cells were cultured in 1:1 mixture of Ham’s F12 Medium and Dulbecco’s Modified Eagle’s Medium (DMEM), which was supplemented with 2.5 mM l-glutamine, 0.3 mg/ml G418 and 10% FBS, 1% penicillin and streptomycin. All the cells were maintained in a humidified incubator at 37 °C with 5% CO_2_. When reaching approximately 80% confluence, cells would be passaged by trypsinization. The detached cells were seeded into a new flask at the density of 2.5 × 10^3^ cells/cm^2^.

To observe the effect of TGF-β1 on LHX9 expression, U2OS and Saos-2 cells were cultured without or with 1, 5, 10 ng/ml TGF-β1 for 24 h, or with 5 ng/ml TGF-β1 for 24, 48, and 72 h, then they were collected for subsequent RT-qPCR and Western blot analysis. To inhibit TGF-βR1 signaling, U2OS and Saos-2 cells transfected with shNC or shFRS2 were cultured in the absence or presence of TGF-βR1 inhibitor (SB431542) for 48 h, then they were collected for subsequent Western blot analysis. To look for new regulators of TGF-βR1 on transcriptional level, U2OS cells transfected with shNC or shFRS2 were cultured without or with transcription inhibitor (ActD) for 24 h, then they were collected for subsequent RT-qPCR analysis.

### Let-7c mimic, inhibitor and controls

*Let*-*7c* mimic and inhibitor were ordered from Genepharma (Shanghai). The sequence of *let*-*7c* mimic: 5′-UGAGGUAGUAGGUUGUAUGGUU-3′; negative control miRNA: 5′-CAGUACUUUUGUGUAGUACAA-3′. The sequence of *let*-*7c* inhibitor: 5′-AACCAUACAACCUACUACCUCA-3′; negative control miRNA: 5′-UUUGUACUACACAAAAGUACUG-3′. *Let*-*7c* mimic, inhibitor and controls were transfected into U2OS and Saos-2 cells by lipofectamine 3000.

### Plasmid construction and transfection

ShRNA targeting *LHX9*, *beta*-*catenin*, *FRS2* and negative control shRNA (shNC) were synthesized in Genepharma and subcloned into pLKO.1 vector. Sequence-verified plasmids were then transfected into HEK293T cells with psPAX2 packaging plasmid and pMD2.G enveloping plasmid by lipofectamine 3000 to generate shRNA-containing lentivirus, respectively. Titer-determined lentivirus was then used to infect U2OS, Saos-2 cells to obtain *LHX9/beta*-*catenin/FRS2*-knockdown cells.

### RNA extraction and quantitative real-time PCR

Total RNA was collected from OS tissues or cell lines that were lysed and homogenized in TRIzol reagent. cDNA was generated from total RNA by SuperScript IV First-Strand Synthesis System with oligo dT(20) primers. qPCR was performed using SYBR Green qPCR Master Mix on CFX96 Touch Deep Well Real-Time PCR Detection System (Bio-Rad). The relative expressions of *LHX9*, *TGF*-*beta 1* and *TGF*-*beta R1* mRNAs were normalized to that of *GAPDH* mRNA. All experiments were performed in triplicate.

### Western blot

After quantification by BCA method, cell lysate samples containing 50 μg of protein were fractioned by SDS-PAGE and transferred to PVDF membrane (Millipore Corp, Bedford, MA), which was then blocked by 10% BSA in Tris-buffered saline with 0.1% Tween 20 (TBST) for 1 h. After that, the PVDF membrane was rinsed and then probed with the primary antibodies against respective target proteins at 4 °C overnight: anti-LHX9 antibody (1:1000, Abcam-ab224357), anti-BMP4 antibody (1:1000, Abcam-ab39973), anti-β-catenin antibody (1:10,000, Abcam-ab32572), anti-COL1A antibody (1:2000, Abcam-ab34710), anti-MMP-1 antibody (1:1000, Abcam-ab137332), anti-Snail-1 antibody (1:1000, Abcam-ab53519), anti-Slug-1 antibody (1:1000, Abcam-ab106077), anti-Twist1 antibody (1:1000, Abcam-ab49254), anti-MMP-9 antibody (1:1000, Abcam-ab73734), anti-TGFβR1 antibody (1:2000, Abcam-ab31013), anti-FRS2 antibody (1:1000, Novus Biologicals-462910), anti-Smad2 (L16D3) antibody (1:2000, Cell Signaling Technology-3103S), anti-phospho-Smad2 (Ser465/467) (138D4) antibody (1:2000, Cell Signaling Technology-3108S), anti-GAPDH (D16H11) XP antibody (1:5000, Cell Signaling Technology-5174S) and anti-β-actin (13E5) antibody (1:5000, Cell Signaling Technology-2128S). After washing, these PVDF membranes were then incubated with the appropriate secondary antibodies for 1 h. After TBST washing, the blots were detected with ECL substrate and imaged by the GelDoc EZ imaging system (Bio-Rad).

### Co-immunoprecipitation

48 h post the transfection, U2OS or Saos-2 cells were collected, rinsed by cold PBS and lysed in 1× cell lysis buffer (20 mM Tris (pH 7.5), 150 mM NaCl, 1 mM EDTA, 1% Triton X-100, 2.5 mM Sodium pyrophosphate, 1 mM β-glycerophosphate, 1 mM Na_3_VO_4_, 1× Proteinase inhibitor cocktail) by rotation at 4 °C for 1 h. After centrifugation at 12,000 rpm, 4 °C for 10 min, the supernatants were collected and transferred to new tubes. 50 μl clear supernatant was directly used as cell lysates to perform Western blot experiment, and the left part was then incubated with anti-Myc tag antibody by gently rotation at 4 °C for 4 h. Next, 40 μl of pre-blocked Protein A/G conjugated agarose beads were added, followed by another 2 h of incubation. After centrifugation and washing three times of the beads, protein samples were eluted by 1× reducing loading buffer and subjected to Western blot. The protein on PVDF membrane was probed by anti-Flag tag antibody (1:5000). For the cell lysate samples, the protein on PVDF membrane was probed by anti-Myc tag antibody (1:3000), anti-Flag tag antibody (1:5000) or anti-β-catenin antibody (1:10,000), respectively.

### Hematoxylin and eosin (HE) staining and analysis

Mouse lung tissues were dissected out and fixed with 4% paraformaldehyde for overnight. After that, the fixed tissue samples were embedded in paraffin, sectioned into pieces with the thickness of 6 μm. The sections were then dehydrated with xylol and different ethanol concentrations, followed by brief washing in distilled water and cell nuclei staining with hematoxylin solution for 10 min. After rinsing in running water for 5 min, the stained samples were differentiated in 0.1% HCl-ethanol for 30 s. After that, they were rinsed in running water for 1 min, blued in PBST for 1 min, rinsed in running water for another 1 min and washed in 95% ethanol for 10 s. Samples were then counterstained with Eosin staining solution for 2 min. HE-stained sections were dehydrated through 95% ethanol and 2 changes of absolute ethanol, cleared in 2 changes of xylol for 5 min each time. Afterwards, the sections were mounted on fluorescence microscopy (IX-51, Olympus) for imaging. 5 randomly selected images were taken for each specimen. Data were collected from at least three independent experiments.

### Cell proliferation by CCK-8 assay

Cell proliferation was determined using a CCK-8 kit following the manufacturer’s instructions. U2OS or Saos-2 cells were seeded into 96-well plates in quintuple and cultured for indicated time. Then, the old medium was replaced by fresh medium containing 10% CCK-8 and then the cells were incubated for another 3 h. An OD absorbance at 490 nm was measured by a micro-plate reader (Bio-Tek).

### Cell colony formation assay

12-well plate was pre-coated with a layer of solidified medium containing 0.8% agarose, then 5 × 10^3^ U2OS or Saos-2 cells were plated on this layer in 1.5 ml culture medium supplemented with 0.4% agarose. Two weeks later, cell colonies were fixed by 4% paraformaldehyde and stained with 0.1% crystal violet solution. After washing by PBS, the whole well was captured and the number of viable colonies larger than 0.1 mm was calculated with Image J software (NIH, Bethesda, MD, USA).

### Scratch wound healing assay

U2OS or Saos-2 cells were pre-seeded in 24-well plates at the density of 2.5 × 10^3^/well. 24 h later, a new 1-ml pipette was used to gently and slowly scratch the cell monolayer across the center of the wells, the detached cells were gently washed away with culture medium. After replenishing the wells with fresh medium, the gap in the monolayer was captured on an inverted microscopy (Olympus IX71, Shinjuku, Tokyo, Japan). Scratched cell monolayer was then cultured for another 24 h, followed by washing with PBS and fixing with 4% paraformaldehyde. The gap in the monolayer was captured again on the inverted microscopy. 5 randomly selected images were taken for each gap in one well, which were analyzed by Image J software (NIH, Bethesda, MD, USA). Data were collected from at least three independent experiments,

### Cell invasion assay using transwell system

For transwell invasion assay, Transwell chambers (Corning, Lowell, MA, USA) were coated with Matrigel (BD Biosciences, San Diego, CA, USA). 5 × 10^4^ U2OS or Saos-2 cells in 500 μl culture medium without FBS were seeded in Transwell inserts (Costar, Corning, NY, USA) and the lower chambers were filled with 500 μl complete medium as a chemoattractant. After incubation for 15 h, cells that have migrated to the lower chamber were washed by PBS, fixed by 4% paraformaldehyde and stained with 0.1% crystal violet. Stained cells were then imaged on an IX71 inverted microscope (Olympus IX71, Shinjuku, Tokyo, Japan) and five random fields per well were captured. Cell number per well was calculated with the captured images using Image J software (NIH, Bethesda, MD, USA).

### Dual-luciferase reporting assay

Dual-luciferase reporter assay was performed by co-transfecting firefly luciferase reporter plasmid containing *TGF*-*beta R1* mRNA (pmirGLO-*luc2* firefly luciferase-TGF-βR1) and *let*-*7c* or control miRNA into 293T cells by lipofectamine 3000. The *Renilla* luciferase gene in pmirGLO vector provided normalization reference. 48 h after transfection, the cells were lysed and processed for subsequent luciferase activity measurement with the Dual Luciferase Assay Kit according to the manufacturer’s instructions. Firefly and *Renilla* luciferase activities were measured by a plate reader (NEO, Bio-Tek, USA) and normalized to *Renilla* luciferase data.

### Tumor models in nude mice

Nude mice were purchased from Shanghai SLAC Laboratory Animal Co. Ltd. and were maintained in pathogen-free facilities at Central South University. All animal experiments used mice with matched age and sex, and they were randomly allocated to experimental groups. All the animal experiments were approved by the Animal Care and Use Committee (ACUC) of Central South University. The maximal tumor volumes were in accordance with the guide of ACUC.

To establish a tumor growing model, shNC- or sh*LHX9*-transfected 143B cells were trypsinized, washed by PBS and filtered through a 40-μm cell strainer. After cell density measurement, 1 × 10^6^ cells were transplanted to 6-week-old nude mice via subcutaneous injection. The tumor growth was monitored by measuring the tumor volume every 5 days with a vernier caliper. Tumour volume was calculated as length × width × height. Mice with tumour size larger than 20 mm at the longest axis were euthanized for ethical consideration. After 4 weeks, the tumor tissues were obtained from euthanized mice, imaged and weighted.

To establish a tumor metastasis model, shNC- or sh*LHX9*-transfected 143B cells were washed twice with PBS and filtered through a 40-μm cell strainer. After that, 2 × 10^6^ cells were intravenously injected into nude mice at age of 6 weeks. To assess the tumour multiplicity in the lung, nude mice were euthanized at day 36 after tumour inoculation and the lung tissues were digested out. Tumors grown in the lung were imaged and tumor number was counted.

### Statistical analysis

All experiments were performed for three times, with one representative experiment shown. Data were represented as mean ± standard deviation (SD). Statistical analysis was performed by GraphPad Prism 7 (GraphPad Software, Inc.). Unpaired two-tailed Students’ *t*-test was used to compare the difference between two groups. One-way analysis of variance (ANOVA) followed by Tukey post hoc test was used for multiple comparison. Statistical significance was determined as indicated in the figure legends. *, **, and *** denoted significance at 0.05, 0.01, and 0.001 level, respectively.

## Supplementary information

**Additional file 1: Figure S1.** The relative expression levels of *LHX1*–*9* and knockdown efficiencies of sh*LHX9*, sh*beta*-*catenin* and sh*FRS2* in OS cells. (A) The relative expression levels of *LHX1*–*9* were measured by qRT-PCR in hFOB1.19 and U2OS. *LHX1*–*9* mRNA levels were normalized to *GAPDH* mRNA (n = 3). (B) U2OS cells or Saos-2 cells were un-transfected or transfected with negative control shRNA (shNC) or shRNA targeting *LHX9* (sh*LHX9*), *beta*-*catenin* and *FRS2,* respectively, then they were cultured for 48 h. The expression of LHX9, beta-catenin and FRS2 in blank control cells, sh*NC*- or sh*LHX9*-transfected cells was measured by Western blot. GAPDH was used as the loading control.

## Data Availability

All data generated or analyzed during this study are included in this published article.

## Abbreviations

FGF: fibroblast growth factor
FGFR: fibroblast growth factor receptor
LXH9: LIM homeobox 9
LMO: LIM only proteins
OS: osteosarcoma
BMP4: bone morphogenetic protein 4
COL1A1: collagen, type I, alpha 1
Twist1: twist-related protein 1
MMP-1: matrix metallopeptidase 1
MMP-9: matrix metallopeptidase 9
FRS2: fibroblast growth factor receptor substrate 2
TGFβ: tumor growth factor-β
TGFβR1: tumor growth factor-β receptor 1
TCF4: transcription factor 4
Hh: hedgehog signaling
RANK: receptor activator for nuclear factor-κB
PI3K: phosphatidylinositol 3-kinase
mTOR: mammalian target of rapamycin
ATCC: American Type Cell Culture
RT-qPCR: real-time quantitative PCR
shRNA: short hairpin RNA
miRNA: microRNA
HE: hematoxylin–eosin
DMEM: Dulbecco’s Modified Eagle’s Medium
