# Supplementary figures and images for: FGF-induced LHX9 regulates the progression and metastasis of osteosarcoma via FRS2/TGF-β/β-catenin pathway
Source: Cell Div. 2019 Nov 25;14:13. doi: 10.1186/s13008-019-0056-6 (PMC6876112; doi:10.1186/s13008-019-0056-6)

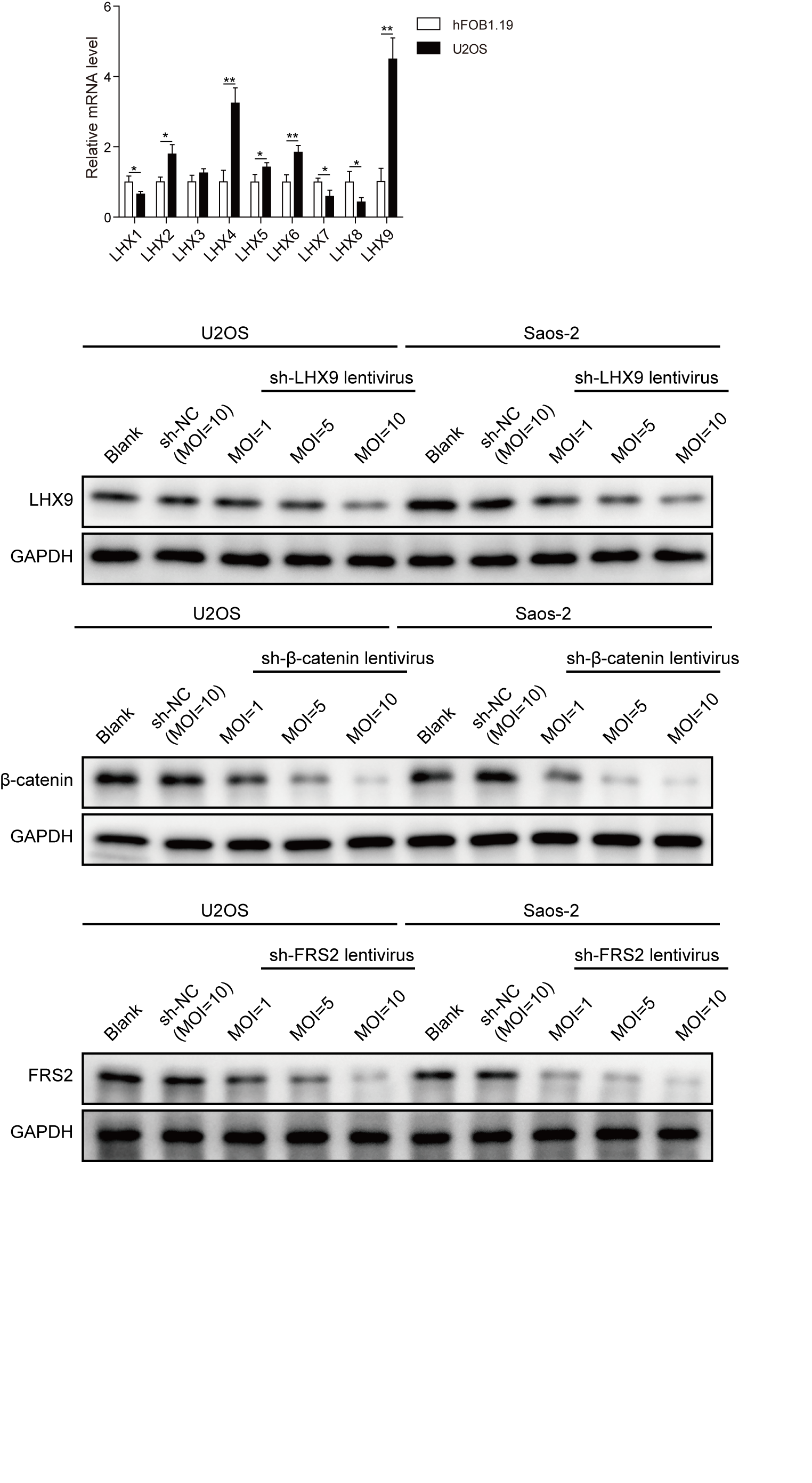

Supplement: Supplementary file 1 — Additional file 1: Figure S1. The relative expression levels of LHX1–9 and knockdown efficiencies of shLHX9, shbeta-catenin and shFRS2 in OS cells. (A) The relative expression levels of LHX1–9 were measured by qRT-PCR in hFOB1.19 and U2OS. LHX1–9 mRNA levels were normalized to GAPDH mRNA (n = 3). (B) U2OS cells or Saos-2 cells were un-transfected or transfected with negative control shRNA (shNC) or shRNA targeting LHX9 (shLHX9), beta-catenin and FRS2, respectively, then they were cultured for 48 h. The expression of LHX9, beta-catenin and FRS2 in blank control cells, shNC- or shLHX9-transfected cells was measured by Western blot. GAPDH was used as the loading control. [file 13008_2019_56_MOESM1_ESM.tif]
